# Recent Advances in Urinary Tract Reconstruction for Neuropathic Bladder in Children

**DOI:** 10.12688/f1000research.7235.1

**Published:** 2016-02-22

**Authors:** Roberto I. Lopes, Armando Lorenzo

**Affiliations:** 1 Division of Urology, The Hospital for Sick Children, Toronto, Ontario, Canada; 2 Department of Surgery, University of Toronto, Toronto, Ontario, Canada

**Keywords:** urinary reservoirs, continent, urinary incontinence, urinary bladder, neuropathic, fecal incontinence

## Abstract

Neuropathic bladder usually causes several limitations to patients’ quality of life, including urinary incontinence, recurrent urinary tract infections, and upper urinary tract damage. Its management has significantly changed over the last few years. The aim of our paper is to address some salient features of recent literature dealing with reconstructive procedures in pediatric and adolescent patients with lower urinary tract dysfunction.

## Introduction

Spinal dysraphism, including myelomeningocele, represents one of the most common permanently disabling birth defects in the United States, with an incidence of 30 cases in 100,000 live births ^1^. “More than 90% of patients with spina bifida have resultant neuropathic bladder dysfunction, which can manifest as urinary incontinence, recurrent urinary tract infections and—in the most severe cases—upper urinary tract damage” ^2^. Unfortunately, some degree of renal impairment is common, affecting up to 30% of adolescents with the condition ^3^. Although most patients can be managed with medication (e.g. anticholinergics) and clean intermittent catheterization, lower urinary tract reconstructive surgery has been introduced and modified over the last few decades to address incontinence and prevent upper tract decompensation. Despite perceived benefits and after a fairly rapid uptake, the estimated number of augmentation cystoplasties performed in children in the United States has now decreased by 25% in the 2000s. The cause for this change is likely multifactorial, including better or earlier introduction of optimal medical management, but ultimately reflects an important change in practice patterns in the United States and may mirror trends in other parts of the world. It is tempting to consider the surgeries’ risk profile—with up to 30% of patients having a potential complication during hospitalization after augmentation cystoplasty—and the known long-term consequences of this procedure as the driving force behind this trend ^4^.

When considering any surgical intervention in patients with neuropathic bladder, evaluation of the patient’s clinical status according to a risk-stratified inventory is advised: one must a) confirm that the upper tracts are stable without new dilation, increasing renal echogenicity, or deteriorating corticomedullary differentiation; b) assess whether the child has been experiencing urinary tract infections (UTIs); and c) determine if urinary incontinence is becoming a concern that the child wishes to have addressed.

Urodynamic or videourodynamic evaluations have proven to be of great value in quantifying bladder dysfunction, helping guide therapy for socially unacceptable incontinence and/or potential renal insults, as well as evaluating the outcome of resulting procedures and interventions. Importantly, a significant proportion of patients with spina bifida have reduced bladder capacity with different degrees of impaired compliance. The most worrisome situation, a “high-pressure” bladder, is characterized by increased leak point pressure, reduced bladder compliance, and detrusor overactivity, a situation that—if untreated—often leads to complications down the road. A detrusor leak-point pressure (DLPP) >40 cm H _2_O, a bladder compliance of <9 mL/cmH _2_O, and evidence of hypercontractile detrusor are all factors that carry some value in predicting the risk of upper urinary tract dysfunction in children with neuropathic bladder ^5^.

 First-line therapy for reduced bladder capacity and/or high-pressure bladder is anticholinergic medication, usually coupled with clean intermittent catheterization. If this approach fails or is not tolerated by the patient, second-line options include a variety of procedures such as botulinum toxin injection, electrical stimulation therapy, and bladder autoaugmentation ^6^. One exciting option is the direct injection of botulinum toxin in the detrusor muscle as a means to quench detrusor overactivity and improve compliance. Over the past few years, this option has gained popularity ^7, 8^. Intra-detrusor OnabotulinumtoxinA (OnabotA) injections have been selectively offered at our institution for cases in which maximal anticholinergic therapy failed or was not tolerated. Thus far, it has shown significant improvement in symptoms, bladder capacity, and compliance, effectively avoiding or delaying the need for augmentation at the expense of regular procedures to deliver the medication ^9^.

 In refractory cases, bladder neck reconstruction (BNR), bladder augmentation, continent diversion (CD), and bladder neck closure (BNC) are offered, with the goal of creating a large capacity and highly compliant reservoir that can be easily accessed (for catheterization) without leakage at expected volumes for age. These surgical approaches may be necessary in 5% to 20% of patients ^10, 11^. Additional procedures are often required. For example, creation of a catheterizable channel (with appendix or reconstructed, tubularized bowel) is offered as a means for providing more convenient access and/or a reliable entry point when catheterization per urethra is difficult or impossible. Similarly, bilateral high-grade vesicoureteral reflux in patients with neuropathic bladder can persist after bladder augmentation in up to half of patients. Many of these develop pyelonephritis during follow-up, even while taking antibiotic prophylaxis. Therefore, at the time of bladder augmentation for noncompliant neuropathic bladder, concomitant anti-reflux surgery should be considered for children with high-grade reflux ^12^. In severe cases of high-pressure non-compliant bladders that are causing renal decompensation, especially in the setting of kids with severe disabilities who would require constant care and assistance, and for whom an easy, failsafe method of bladder management is preferred, a vesicostomy can be used as a temporizing factor for some and a permanent solution for others.

Within this framework, herein we will discuss some salient features of recent literature dealing with reconstructive procedures in pediatric and adolescent patients with lower urinary tract dysfunction.

## Augmentation cystoplasty

Bladder augmentation and related urinary diversion techniques aim to dramatically reduce pressure to the upper tracts, prevent further renal damage, and aid in continence. However, these procedures have several drawbacks. For patients who undergo ileal loop urinary diversion, limitations include altered body image, management of an external appliance, and the potential for recurrent pyelonephritis, nephrolithiasis, and delayed anastomotic stricture. Most patients who undergo bladder augmentation require intermittent catheterization, with its own set of challenges. More importantly, long-term complications of bladder augmentation include metabolic derangement, bladder stones, recurrent UTI, bladder perforation, and an ill-defined increased risk of malignancy. The choice between urinary diversion and bladder augmentation is complex for surgeons and patients. Surgeon factors include comfort with the surgical technique and resources for subsequent management. For patients and their families, important considerations include body image, social and cultural issues, ability to perform intermittent catheterization, anticipated compliance with long-term follow-up, need for indefinite monitoring, and unknown problems as decades go by exposing intestinal epithelium to urine.

In one of the largest studies comparing these two techniques, bladder augmentation was performed in an estimated 3403 patients and ileal loop diversion in 772 patients with spina bifida between 1998 and 2005. Patients undergoing bladder augmentation were younger (mean age 16 vs. 36 years, p <0.001), more often male (52% of bladder augmentations vs. 43% of urinary diversions, p = 0.02), and privately insured (46% vs. 29%, p <0.001) compared to those undergoing urinary diversion. “Furthermore, patients undergoing urinary diversion required more health care resources, with significantly longer hospital stays, higher total charges and more use of home health care after discharge home” ^13^. This highlights important differences in demographics and health care resource utilization between populations exposed to different surgical strategies and might indicate that children getting urinary diversions are generally sicker at baseline evaluation.

Children who underwent augmentation cystoplasty identified in the Pediatric Health Information System over a decade were assessed to evaluate their surgical outcomes. A total of 2831 augmented patients were assessed and 10-year cumulative incidence ranges for the following outcomes and procedures were achieved: bladder rupture (2.9–6.4%), small bowel obstruction (5.2–10.3%), bladder stones (13.3–36.0%), pyelonephritis (16.1–37.1%), and need for cystolithopaxy (13.3–35.1%) and re-augmentation (5.2–13.4%). “The development of chronic kidney disease was strongly associated with a diagnosis of lower urinary tract obstruction (HR 13.7; 95% CI 9.4–19.9). Bladder neck surgery and stoma creation at time of augment were associated with an increased hazard of bladder rupture (HR 1.9; 95% CI 1.1–3.3) and bladder stones (HR 1.4; 95% CI 1.1–1.8) respectively”. Results from this large cohort can be used to counsel patients and families about expectations for surgical intervention, including those that carry important morbidity, such as bladder perforation ^14^. Finally, the risk of malignancy development has been of concern to many but appears to be <5% ^15, 16^. Obviously, this may dramatically change as the patient population ages and is exposed to carcinogenic stimuli (such as smoking and chronic irritation). The issue of malignancy should be further considered in all patients with neuropathic bladder, with or without augmentation, and can be the end-result of catheterization, infections, and/or colonization. As recently reported by Husmann *et al.*, other factors (such as immunosuppression) can drive the risk of carcinogenesis in a population already at risk due to their lower urinary tract dysfunction ^15^.

With the widespread interest in minimally invasive surgery, robotic augmentation and appendicovesicostomy has been recently described, isolated to centers with extensive expertise. Although feasible, the surgery does appear somewhat cumbersome and time-consuming. The intervention clearly tried to mimic the surgical steps of its open counterpart. For augmentation cystoplasty, the patient is placed in the Trendelenburg position. An umbilical 12 mm trocar is placed, followed by two 8 mm robotic ports and two assistant ports. After pneumoperitoneum creation, the bladder, small bowel, and appendix are assessed. Stay sutures are placed 20 cm apart on the portion of the ileum that will be used for augmentation, 20 cm from the ileocecal junction. Entero-entero anastomosis is performed in an end-to-end fashion followed by mesenteric window closure. For appendiceal dissection, a stay suture in the tip is used and a window is created in the mesentery of the appendix, making sure that its blood supply is preserved. The appendix is detached from the cecum, with the latter closed in two layers. Detrusorotomy is performed after distension of the bladder with saline, and the tip of the appendix is spatulated and anastomosed to the bladder mucosa after a 1 cm opening of its more distal aspect. Appendicovesical anastomosis is performed in a continuous fashion over a 8 Fr feeding tube placed through the appendix, and after that a tunnel to avoid reflux is created by the imbrication of the detrusor muscle over the appendix. The bladder is bivalved to receive the detubularized ileal segment, which is anastomosed as in open augmentation. An 18 Fr Foley catheter is introduced into the bladder before completing the ileovesical anastomosis. The appendix can be exteriorized through the umbilicus or the lower quadrant of the abdomen. The augmented bladder is drained with suprapubic and urethral catheters until ready to start clean intermittent catheterization through the appendicovesicostomy 7–10 days post op ^17– 19^. The reported average operative time was 8.4 hours (range 6–11 hours) and no major intraoperative complications were encountered. Perioperatively, patients required oral analgesia for 24–36 hours, started on a liquid diet after 7.5 hours (range 6–10 hours), went on a regular diet after 24 hours (range 12–36 hours), and were discharged home within 7 days. All patients now have day and night time continence without UTIs, and bladder capacity between 250 and 450 mL. While longer follow-up will be necessary to see if these results are durable, this series demonstrates that robotic alternatives are safe and feasible in the short term, with the possible added benefits of reduced analgesia and recovery time, along with aesthetic benefits. Rightfully, it is important to question all these outcomes against open surgical procedures with less expensive resource utilization and acceptable, well-described, longer-term outcomes.

## Gastric augmentation

Gastric augmentation seemed like a great idea when originally introduced, particularly for patients with chronic renal insufficiency, in which the acid-base balance changes expected from recurrent drainage of acid gastric mucosa outputs would benefit the patient. Unfortunately, after long-term follow-up, complications can be expected in over half of patients. Malignancies have developed in the reservoir in some patients in as little as a decade after gastrocystoplasty ^20^. We currently do not recommend the use of gastric segments for reconstruction of the lower urinary tract due to the high incidence of reoperations and complications. In patients in whom gastric segments were used in the past for lower urinary tract reconstruction, regular surveillance and close follow-up are strongly advocated, even though it is unknown if any preventive or screening strategies are of value.

Metabolic complications with the use of stomach for urinary reconstruction have been previously described. Abnormalities directly related to the secretion of hydrochloric acid by the gastric patch incorporated in the urinary tract include the hematuria-dysuria syndrome, hypochloremic metabolic alkalosis, and hypergastrinemia. The hematuria-dysuria syndrome is a unique complication of the use of gastric tissue for lower urinary tract reconstruction. The secretion of acid can irritate the bladder and urethra, and the reported incidence can be as high as 36%. Severity of symptoms varies, and many cases are relatively minor and can be controlled with H2 histamine blockers, proton pump inhibitors, and increased hydration and frequency of catheterization. In unresponsive cases, removal of gastric tissue is necessary ^20^.

## Biomaterials

The idea of using a readily available “off-the-shelf” material has attracted investigators for decades. Only a few clinical studies have been reported, showing mostly disappointing results, employing biological materials such as dura mater or small intestinal mucosa ^21^. When contrasted with the use of autologous intestinal tissue, these alternatives have not shown significant benefit and can lead to failure or complications.

## Bioengineered bladder

Experimental efforts to construct a tissue-engineered bladder with a scaffold seeded with cultured cells previously obtained from the patient’s bladder have been reported, culminating in a somewhat controversial report of a few patients with myelomeningocele, showing limited application and mostly disappointing results ^22^. The current techniques for autologous cell-seeded biodegradable scaffolds do not appear to improve bladder compliance or capacity in a clinically superior way, and serious adverse events appear to surpass acceptable safety standards ^23^.

An important problem with contemporary approaches to tissue engineering and cell therapy for urinary tract reconstruction is the requirement for invasive tissue biopsies to obtain autologous cells. Aside from the need to accept the morbidity and exposure to anesthesia for such procedure, there is a theoretical problem with employing cells whose genetic and epigenetic footprint may have been altered from development in a diseased organ. As an alternative, a urine-isolated subpopulation of cells with progenitor cell features and the potential to differentiate into several bladder cell lineages can be employed. These urine-derived cells could serve as an alternative source for urinary tract tissue engineering and reconstruction ^24^. The ultimate value is speculative, and animal and human studies are clearly needed. This exciting field is growing at an exponential pace. Even though in clear need of a breakthrough, the promise fuels hope for patients and families.

## Buttons

Buttons might be an alternative to patients who are not amenable to augment/diversion and/or clean intermittent catheterization. Two different types of button can be used: the Mic-Key button (Ballard Medical Products) and the Mini balloon button (Applied Medical Technology). Both are silicone devices. They consist of an internal portion, resembling the tip of a Foley catheter, with an inflatable balloon to create a self-retaining mechanism against the abdominal wall. The external portion comprises a flat button that sits on the skin surface. It contains a valve to prevent leakage unless the drainage adapter is in place. The devices are available in a range of sizes (12–24 Fr) and lengths (0.8–5.0 cm), allowing the catheter to be individualized based on the patient’s characteristics and growth over time. Although an interesting option, there is a paucity of data suggesting superiority over a suprapubic catheter or other forms of diversion. Furthermore, its use does not address problems with capacity or compliance, only access for bladder drainage ^25^.

## Catheterizable channels

The Mitrofanoff principle for creating a continent, catheterizable stoma using the appendix has been a mainstay in the armamentarium of the pediatric urologist to provide access to the bladder either with or without augmentation since its description and popularization in the 1980s ^26^. The use of a transverse tubularized bowel segment, as described by Yang and Monti ^27^, and further modified by Casale ^28^, has now become the preferred option when the appendix is unavailable. Irrespective of how the channel is fashioned, patients and families should be aware about the potential need for reinterventions, which persists even during long-term monitoring ^29^.

**Figure 1.**
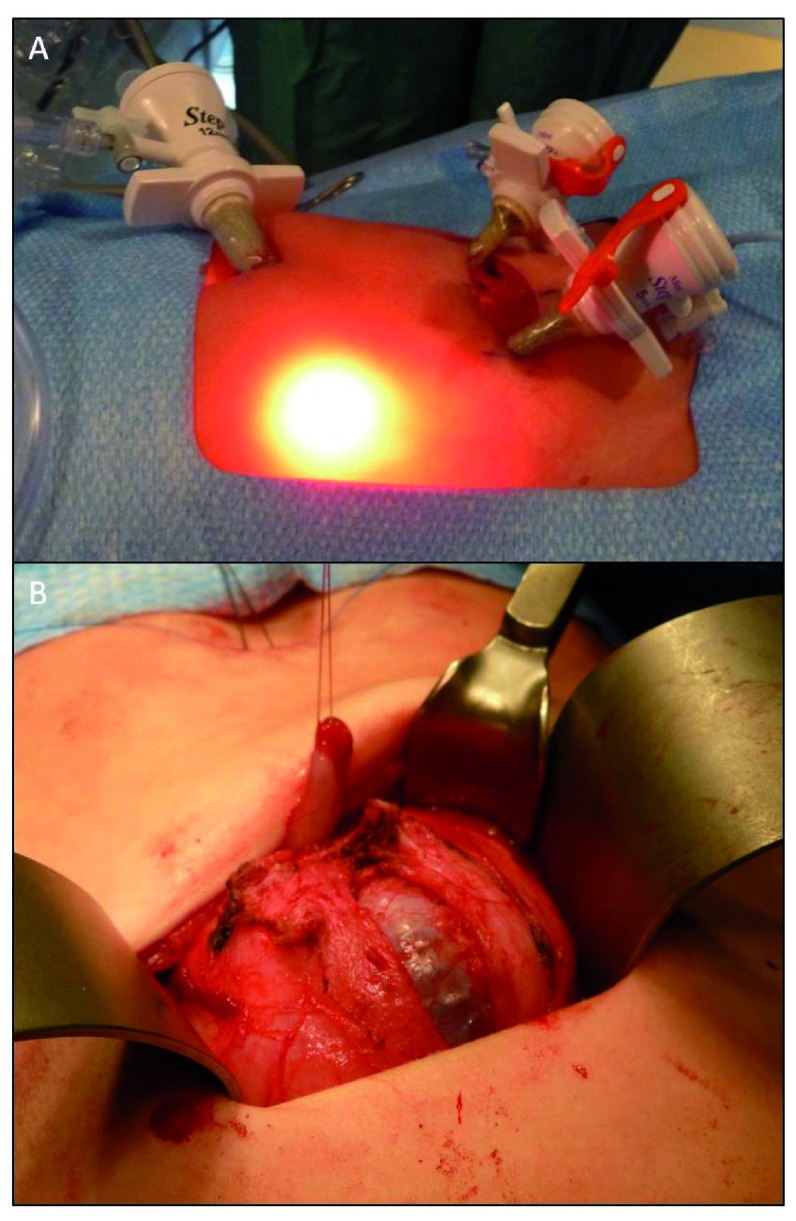
Laparoscopic-assisted Mirofanoff channel: **A**. Laparoscopy (with ports placed in the umbilicus and along the planned Pfannenstiel-type incision) to identify and mobilize the appendix towards the pelvis; **B**. Detrusorotomy and confection of Mitrofanoff channel.

Similar to robotic augmentation cystoplasty, robotic appendicovesicostomy is gaining popularity. The patient is placed in the Trendelenburg supine position, and an 8 or 12 mm camera port is placed at the umbilicus with two 8 mm working ports and one 5 or 12 mm assistant port. The appendix is identified and separated from the cecum with an articulating 55 GIA™ vascular stapler and carefully mobilized on its blood supply. An approximately 4 cm posterior detrusorrhaphy is created, the appendix is laid in the channel, and the bladder is closed over it in an interrupted or running fashion with 3-0 absorbable suture. The bladder is hitched to the abdominal wall and the appendix brought to the umbilical port site, where the stoma is matured. A catheter is left in the appendicovesicostomy channel for approximately 4 weeks ^30^. Comparison of robotic and open appendicovesicostomy revealed no significant difference in the number of acute complications or reoperations between groups. However, the nature and timing of complications differed between groups, being earlier in the robotic approach and later in open surgery ^31– 33^. Statements regarding equivalency or slight (statistically significant) benefit between open and robotic procedures call into question the presence of any clinical benefit when considering resource utilization and expenses. As a bridge, laparoscopic Mitrofanoff has also been reported to have good results, being a feasible, safe, and effective technique associated with low morbidity ^34^. In some centers, including ours, a mixed approach has also been used, employing laparoscopy (with ports placed in the umbilicus and along the planned Pfannenstiel-type incision) to identify and mobilize the appendix towards the pelvis, followed by open detachment from the cecum and anastomosis to the bladder ( Figure 1A and B).

## Slings

Patients presenting urethral intrinsic sphincteric insufficiency (low DLPP) can benefit from surgically increasing resistance as part of urinary tract reconstruction. On comparison, patients undergoing slings with and without augmentation appear similarly successful in achieving improved continence, with patients undergoing augmentation having a longer interval between catheterization and requiring fewer anticholinergics. However, this has to be interpreted with caution, as the patient characteristics in these groups are bound to be different. Health-related quality of life responses revealed that both cohorts were similarly satisfied with the outcomes ^35^. There are some potential differences in outcomes related to the type of procedure performed. For example, patients undergoing Leadbetter-Mitchell procedure plus fascial sling may be less likely to require pads postoperatively than those having a sling alone. Other BNR strategies may be associated with difficulty catheterizing per urethra, thus are often performed in conjunction with a catheterizable channel. Ultimately, this highlights the possibility that some of these procedures are in effect acting as a BNC. In addition, the long-term implications of these procedures are not well defined, and there are evolving challenges that present over time. The best example of such a statement is the disappointing development of problems after BNR without augmentation in a cohort of patients initially reported to enjoy great outcomes. After a mean follow-up of almost 5 years, the updated series described over 50% need for additional continence surgery augmentation cystoplasty, along with a high proportion of vesicoureteral reflux, hydronephrosis, and newly diagnosed or worsening renal scarring ^36^. These findings begin to resemble other cautionary reports addressing issues over prolonged monitoring ^37^, highlighting the need for close monitoring and critical assessment of successful interventions reported after relatively short follow-up.

Of all the described procedures and their variations, bladder neck sling cystourethropexy is a commonly used one to correct relative sphincter deficiency in children with spinal dysraphism. Various modifications of the procedure have been made, but as a common theme they involve circumferential dissection of the bladder neck and proximal urethra. Robotic-assisted laparoscopic placement of a bladder neck sling has been recently performed for two female patients with intrinsic sphincter deficiency but adequate bladder compliance. Both procedures were completed intracorporeally. The mean blood loss was 20 mL. The mean operative time was 189 minutes. No intraoperative or postoperative complications occurred. The mean hospital stay was 3 days (range 2–4). The follow-up ranged from 13 to 22 months. Postoperative studies revealed continued low-pressure, compliant bladders and stable upper tracts. At last follow-up, the two patients were using catheterization without difficulty and were continent ^38^. A step-by-step description of the technique and recommendations on robotic instrumentation have been recently published ^39^.

## Bladder neck injections and reconstruction

Deflux® (Dx/HA) bladder neck injection after slings results in modest and often disappointing results. For example, in one series, dryness was achieved in only 25% of patients after failed sling. Second injections after either failure rarely achieve dryness and are hard to justify ^40^. Other groups have reported that endoscopic treatment of neurogenic urinary sphincter insufficiency with Dx/HA is effective in about half of patients ^41^. In general, failure should call for attention to bladder dynamics, a potential culprit for persistent incontinence, and avoid restricting future interventions solely to the bladder neck.

As previously mentioned for slings, isolated bladder outlet procedures for neurogenic incontinence (including the use of artificial sphincters) portend a poor long-term outcome. During long-term follow-up, these patients often require additional interventions (such as augmentation cystoplasty) despite diligent use of anticholinergic medications and strict catheterization. Unfortunately, preoperative urodynamic evaluation does not appear to predict the need or timing for initial bladder outlet procedure or future augmentation cystoplasty, supporting the dynamic picture presented by neuropathic dysfunction, which can change over time and in response to surgical interventions ^42^.

Recently, robotic Leadbetter bladder neck surgical repair has been reported. The patient is placed supine on a surgical beanbag positioner. A 14 F urethral catheter is placed. Pneumoperitoneum is created. An umbilical 12 mm port for the robotic camera is employed, with two 8 mm and one 12 mm additional working ports. A crescent-shaped incision is made posterior to the bladder to drop the rectum in males or vagina in females. The peritoneum is incised and space of Retzius developed. On the posterior aspect, the rectovesical space is developed, from which point the aforementioned tunnelers are advanced ventrally into the developed space of Retzius. The bladder is dropped anteriorly, and the suspensory puboprostatic ligaments are divided. A dorsal vein stitch is placed before cutting down to the catheter and unroofing the proximal urethra and bladder neck to the level of the interureteric ridge. The ureteral orifices are identified, aided by the intravenous injection of indigo carmine. After exchanging the Foley catheter for a 5 Fr feeding tube, the bladder neck/urethra is retubularized in two layers. Afterwards, the tunneling devices are identified and employed to tightly wrap a 360 degree sling that is subsequently attached to the pubic bone using screws from a hernia tacker ^43^.

Postoperative bladder capacity was reported as adequate and augmentation was not deemed necessary. Mean DLPP was 29 cm H _2_O. Mean operative time was close to 8 hours (range 5:56–12:18), including time for appendicovesicostomies that were also performed for clean intermittent catheterization. Mean length of stay and blood loss were 85.7 hours and 117.8 mL, respectively. Postoperatively, all patients were completely dry on clean intermittent catheterization and anticholinergics. This initial series of robot-assisted appendicovesicostomy with BNR and sling placement demonstrates the procedure to be feasible and safe. Needless to say, there is little theoretical basis to suggest that robotic procedures would be less likely to trigger deterioration during follow-up, thus long-term monitoring is required irrespective of how the bladder neck intervention is carried out.

## Bladder Neck Closure

BNC is often seen as a last resort. It is sometimes necessary to improve quality of life in severe refractory cases. In boys, the bladder is transected just cranial to the prostate, after individualizing the neurovascular bundles. In girls, transection is done between the bladder neck and urethra. The bladder is then mobilized on the dorsal side to the level of the ureteric orifices. The bladder neck and urethral stump are then closed. In order to avoid contact, the bladder stump is fixed ventrally to the pubic bone and urethral stump dorsally. If possible, an omental flap might be brought in between the two stumps ^44^. BNC in conjunction with enterocystoplasty and Mitrofanoff diversion is an effective means of achieving continence in complex cases as a primary or secondary therapy ^45^.

## Artificial urinary sphincter

Another option to address urinary intrinsic insufficiency is the artificial urinary sphincter. Although it provides a good rate of continence, complications are frequent, leading to removal in 20% of cases. Critics highlight the added issue of mechanical dysfunction. Over time, only a limited number of patients can empty the bladder without clean intermittent catheterization, which is touted as one of the great benefits of the intervention ^46^.

## Fecal incontinence

Fecal incontinence has a significant impact on quality of life, leading to loss of self-esteem, social isolation, and depression. Initial management of neurogenic bowel includes stool softeners, bulking agents, and − if these approaches fail or prove insufficient − timed evacuation with suppositories and retrograde enemas. However, self-administering enemas can be challenging for patients with limited dexterity and challenge independence. Surgical options to address this issue include the Malone antegrade continence enema (MACE) and cecostomy button; these allow for direct administration of fluid into the bowel, which is accessed through the abdominal wall.

The “MACE principle” was first introduced in the 1990s ^47^. Significant improvements in quality of life following this procedure have been observed. The intervention follows the Mitrofanoff principle and involves creating an appendicostomy as a conduit for antegrade enema administration. A percutaneous technique, with image-guided insertion of a cecostomy tube for similar antegrade enema administration, was established in 1996.

Laparoscopically assisted MACE is now performed in most pediatric referral centers. A 5 mm scope is inserted through the umbilicus and two ports are placed in the left lower quadrant. The appendix is mobilized, brought to the umbilicus, and fixed to the abdominal wall or right lower quadrant.

For cecostomy button placement, a 20 Fr Foley catheter is placed per rectum and the colon is insufflated with air. A suitable access site overlying the cecum is selected and ultrasound is used to confirm the safety of the access. Two suture anchor needles are used to secure the cecum. The initial tube is placed in one of the two access sites. The tract is dilated to approximately 8 Fr and an 8 Fr Dawson-Mueller Mac-Loc® catheter is placed. Six weeks later, the second portion of the procedure is completed, when the catheter is changed to a Chait Trapdoor™ cecostomy catheter, under fluoroscopic guidance over a wire. General anesthesia is usually required. Yearly exchanges of the cecostomy catheters are recommended. Young children require general anesthesia for the initial tube changes, while older patients tolerate it without any sedation.

Fecal continence rates of up to 85% for MACE and 90% for cecostomy tubes have been reported. Mean length of hospital stay for patients undergoing cecostomy vs. laparoscopically assisted MACE was similar. Complications included stomal pain (23% of patients) and difficulty with catheterizing (19%) following MACE, and difficulty flushing (26%) following cecostomy. There were no significant differences between MACE and cecostomy button with respect to fecal continence or complication rates. Each approach poses unique challenges, suggesting that physicians, patients, and families need to understand the differences to make an individualized choice ^48^.
